# The Notification of Venereal Diseases

**Published:** 1916-09-16

**Authors:** Thomas Barlow, Hubert M. Southwark

**Affiliations:** President. Kingsway House, Kingsway, W.C.; Vice-President Kingsway House, Kingsway, W.C.; Vice-President Kingsway House, Kingsway, W.C.


### THE EDITOR'S LETTER-BOX.

### The Notification of Venereal Diseases.

*To the Editor of* The Hospital.

Sir,—As some misunderstanding seems to have arisen in regard to the attitude of the National Council for Combating Venereal Diseases towards the question of compulsory notification of venereal disease, we desire to point out that we are determined to adhere strictly to the recommendations of the Royal Commission. That body carefully considered this question, and arrived at the conclusion that notification at the present time was impracticable, and might be detrimental to the operation of the measures it advocated.

A great mass of evidence was taken, and the balance was strongly opposed to compulsory methods of this nature. The Commissioners, however, recognised that, when public opinion became more enlightened and adequate facilities for treatment had been provided, "the question of notification should then be further considered." They added that, when these conditions have been fulfilled, it is "possible that . . . notification in some form will be demanded."

We are convinced that this view is sound, and the National Council will, therefore, lend no support to any proposals having for their object the establishment of compulsory notification—proposals which would necessarily lead to controversy at a time when unanimity of effort is essential.—We are, Sir, yours obediently,

Sydenham, President.

Thomas Barlow, } Vice-

Hubert M. Southwark. } Presidents

Kingsway House,
Kingsway, W.C.
September 5, 1916.

